# Potential Role of Transient Receptor Potential Channel M5 in Sensing Putative Pheromones in Mouse Olfactory Sensory Neurons

**DOI:** 10.1371/journal.pone.0061990

**Published:** 2013-04-16

**Authors:** Arisa Oshimoto, Yoshihiro Wakabayashi, Anna Garske, Roberto Lopez, Shane Rolen, Michael Flowers, Nicole Arevalo, Diego Restrepo

**Affiliations:** 1 Department of Cell and Developmental Biology, Neuroscience Program, and Rocky Mountain Taste and Smell Center, University of Colorado School of Medicine, Aurora, Colorado, United States of America; 2 Laboratory of Neurobiology, National Institute of Agrobiological Sciences, Tsukuba, Japan; Duke University, United States of America

## Abstract

Based on pharmacological studies of chemosensory transduction in transient receptor potential channel M5 (TRPM5) knockout mice it was hypothesized that this channel is involved in transduction for a subset of putative pheromones in mouse olfactory sensory neurons (OSNs). Yet, in the same study an electroolfactogram (EOG) in the mouse olfactory epithelium showed no significant difference in the responses to pheromones (and odors) between wild type and TRPM5 knockout mice. Here we show that the number of OSNs expressing TRPM5 is increased by unilateral naris occlusion. Importantly, EOG experiments show that mice lacking TRPM5 show a decreased response in the occluded epithelia to putative pheromones as opposed to wild type mice that show no change upon unilateral naris occlusion. This evidence indicates that under decreased olfactory sensory input TRPM5 plays a role in mediating putative pheromone transduction. Furthermore, we demonstrate that cyclic nucleotide gated channel A2 knockout (CNGA2-KO) mice that show substantially decreased or absent responses to odors and pheromones also have elevated levels of TRPM5 compared to wild type mice. Taken together, our evidence suggests that TRPM5 plays a role in mediating transduction for putative pheromones under conditions of reduced chemosensory input.

## Introduction

OSNs express one olfactory receptor type among a repertoire encoded by ∼1,400 genes [1], [2]. Odorant binding initiates Gα_olf_-mediated activation of adenylyl cyclase type III (ACIII) [3]–[5] with subsequent cAMP-mediated activation of the cyclic-nucleotide gated channels [6], [7] eliciting an increase in intracellular Ca^2+^ that *may* lead to opening of the Ca^2+^-gated Cl^−^ channels [8]–[10] depolarizing the membrane potential [11]–[15]. Results of experiments with TRPM5 immunohistochemistry and GFP expression in an animal line where the TRPM5 promoter drives GFP expression indicated that a subset of OSNs express the Ca^2+^ gated TRPM5 channel in olfactory cilia [16]. Although in taste cells TRPM5 plays a critical role for detecting sweet/umami/bitter compounds by directly depolarizing the membrane potential [17], [18], the signal transduction role of TRPM5 in OSNs remains largely unknown [14].

TRPM5-expressing OSNs display a zonal expression pattern, and are abundantly found in the lateral and ventral regions and sparsely represented in the septum in the olfactory epithelium (OE) [16]. This distribution may hint at the functional role of TRPM5 as computer-simulated airflow within rat olfactory turbinates suggests that inhaled air travels 10 times slower in the lateral and ventral regions where a number of TRPM5-expressing OSNs are found, when compared to the computer-simulated airflow in the medial regions [19]. Thus this distribution pattern of TRPM5-expressing OSNs within the olfactory turbinate suggests two possible roles for TRPM5. One is that the TRPM5-expressing cells are located in the lateral and ventral regions to detect specific odorant(s) and/or pheromone(s) that take longer to be absorbed into the mucus and so will achieve stimulation in OSNs in regions with slow airflow. Alternately, expression of TRPM5 could achieve enhanced chemical stimulus-induced activity in the OSNs that are exposed to lower odorant/pheromone levels in the slow airflow regions of the nasal cavity.

Previously, EOG recordings in mouse epithelia did not show any difference between wild type mice and TRPM5 knockouts [16]. However, that could be explained by the fact that if the population of the OSNs that express TRPM5 was already small removing that population by a genetic deletion might not have a large impact on the EOG. Importantly, studies have shown that olfactory transductory proteins increase their expression levels following naris occlusion [20]–[22]. We reasoned that, if TRPM5 plays a role in olfactory or pheromone transduction, its expression might be increased in the epithelia ipsilateral to the occluded naris. In this study, we asked whether the expression of TRPM5 is increased on the occluded side of the epithelia, and whether the genetic deletion of TRPM5 affects the EOG responsiveness to odorants or putative pheromones in the occluded side of the epithelia. In complementary experiments we characterized TRPM5 expression in CNGA2 knockout mice where almost all OSNs lack the ability to generate action potentials and EOG upon odorant stimulation [23], [24].

## Materials and Methods

### Animals

Wild type (C57BL/6), TRPM5-GFP, and TRPM5 knockout mice were bred in the animal facilities of the University of Colorado Anschutz Medical Campus. TRPM5-GFP mice (kindly provided by Dr. Robert Margolskee) contain a TRPM5-GFP construct including 11 kb of mouse TRPM5 5′-flanking sequence, TRPM5 exon 1 (untranslated), intron 1, the untranslated part of exon 2, and eGFP [25]. We used the polymerase chain reaction (PCR) to genotype the offspring for the presence of the GFP-encoding sequence. We crossed the heterozygous CNGA2 knockout female mice (kindly provided by Dr. John Ngai, University of California, Berkeley) [23] with the TRPM5-GFP mice to obtain CNGA2 knockout/TRPM5-GFP mice. TRPM5 knockout mice (kindly provided by Dr. Margolskee) [17] had a deletion of 2.4 kb of the *TRPM5* gene's 5′-flanking region containing the promoter and exons 1–4, including the translation start site within exon 2. All procedures conducted on the animals were in compliance with the University of Colorado Anschutz Medical Campus Institutional Animal Care and Use Committee.

### Unilateral naris occlusion

At postnatal day 5 (PD5) mice were anesthetized by hypothermia and then had the right naris occluded by high temperature cautery [26]. The left naris was open as an internal control. Naris occlusion was tested by addition of soapy water to the occluded naris. These mice were either transcardially perfused with fixative for the immunohistochemistry or euthanized with CO_2_ for the quantitative real-time PCR (q-RTPCR) and EOG experiments.

### Immunohistochemistry

All animals were sacrificed between PD30–60. Animals were anesthetized with sodium pentobarbital (60 mg/kg) and perfused transcardially with 0.9% saline followed by 0.1 M phosphate buffer (PB) (28.75 mM NaH_2_PO_4_, 75 mM Na_2_HPO_4_) containing 3% paraformaldehyde (PFA), 18.75 mM L-lysine monohydrochloride, and 0.23% sodium m-periodate (pH 7.3–7.4). The olfactory epithelium (OE) and the olfactory bulbs (OB) were dissected out and post-fixed in the same fixative for 2 hrs. Cryoprotection was carried out in 30% sucrose overnight at 4°C. OE and OB were cryosectioned coronally at 14 and 18 µm respectively. The sections were rinsed and incubated in 0.1 M PBS, 0.1% Triton X-100 in 0.1 M PBS (0.1% PBT), and blocked in 5% donkey normal serum and 1% bovine serum in 0.1 M PBS. The sections were incubated in primary antibodies for 18 hrs to 48 hrs at 4°C. We used the following primary antibodies: chicken anti-GFP IgY antibody (Avēs Labs, Inc., lot # 0609FP10) at a dilution of 1∶1000, anti TRPM5 polyclonal rabbit antibody provided by Dr. Robert Margolskee at a dilution of 1∶500, anti anoctamin 2 (ANO2) rabbit polyclonal antibody provided by Dr. Thomas Jentsch used at a dilution of 1∶1000 and goat anti olfactory marker protein (OMP, Wako, Code No. 019­22291) at a dilution of 1∶1000. After washing in 0.1 M PBS, the sections were incubated in Alexa 488-conjugated anti-chicken secondary antibody (1∶400, Invitrogen) for 1 hr at RT. Sections were washed in 0.1 M PBS three times and coverslipped with Fluoromount-G (SouthernBiotech). OB images were obtained using a Nikon Eclipse E600 equipped with epi-fluorescence and a Nikon DS Qi1Mc camera (Nikon Instruments Inc.). OE sections were imaged using a SP5 Leica confocal microscope (Leica). As a control, mice that did not carry GFP construct were used and they showed no immunoreactivity against GFP (not shown). The antibodies against TRPM5 and ANO2 label the ciliary layer (and cell bodies to a smaller intensity) in wild type but not in knockout mice [10], [16].

### Data analysis

#### Olfactory epithelium

**Figure 1:**
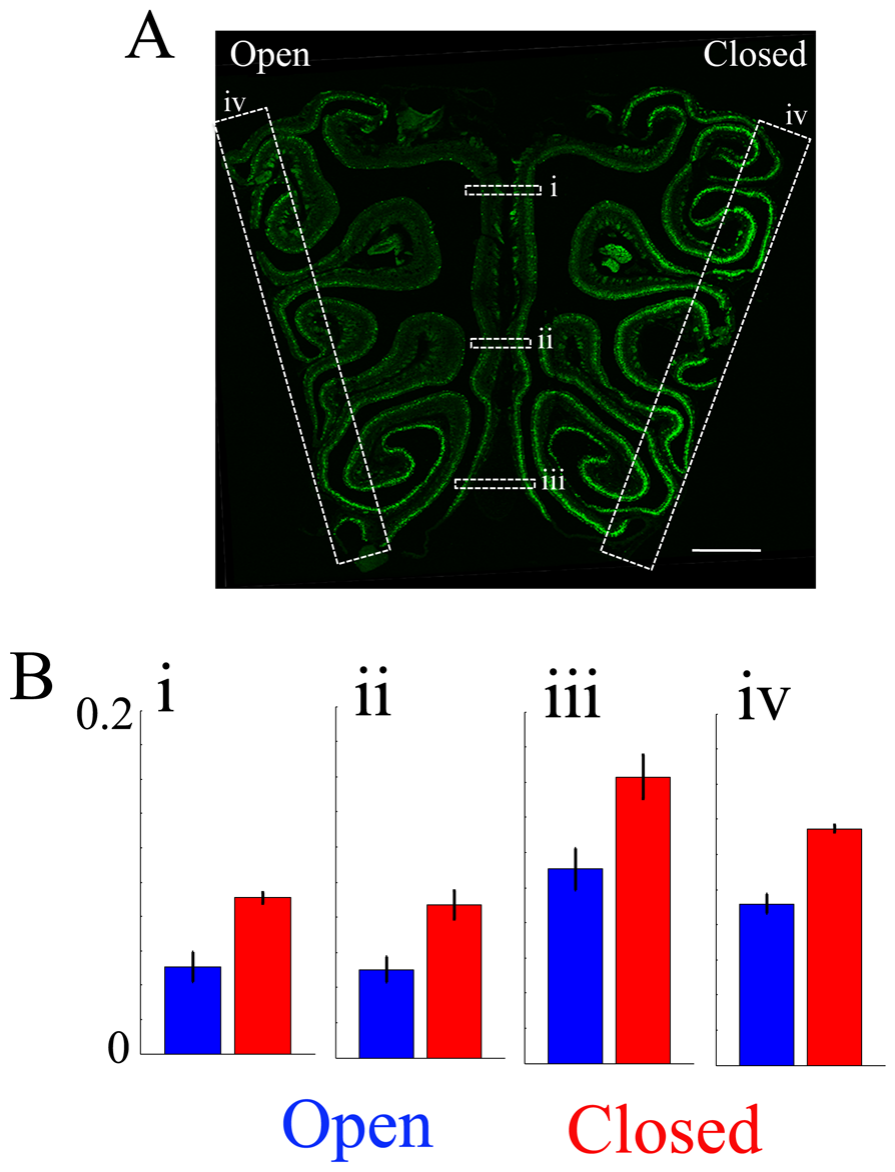
Naris occlusion upregulates GFP immunofluorescence in the occluded side of the OE of TRPM5-GFP mice. A. This panel shows a representative image of a 14 µm coronal section of the OE taken from a naris-occluded animal. The right side in the image is ipsilateral to the occluded naris (Closed) (scale bar  = 500 µm). GFP immunofluorescence is green. B. Averaged GFP immunofluorescence (intensity ranges from 0 to 1, with a gain set so that OSN GFP immunofluorescence ranged from 0 to 0.2). The left side in the image is ipsilateral to the open side of naris (Open). Averaged GFP immunofluorescence intensity in the OE in the septum (i–iii) and the lateral regions (iv) were compared between open and closed sides. Averaged fluorescence intensity was significantly higher in the closed side of epithelium in all locations. (i; *p* = 0.02, ii; *p* = 0.006, iii; *p* = 0.002, iv; *p* = 0.007, p value FDR corrected 0.05, paired t-test, n = 4).

**Figure 2:**
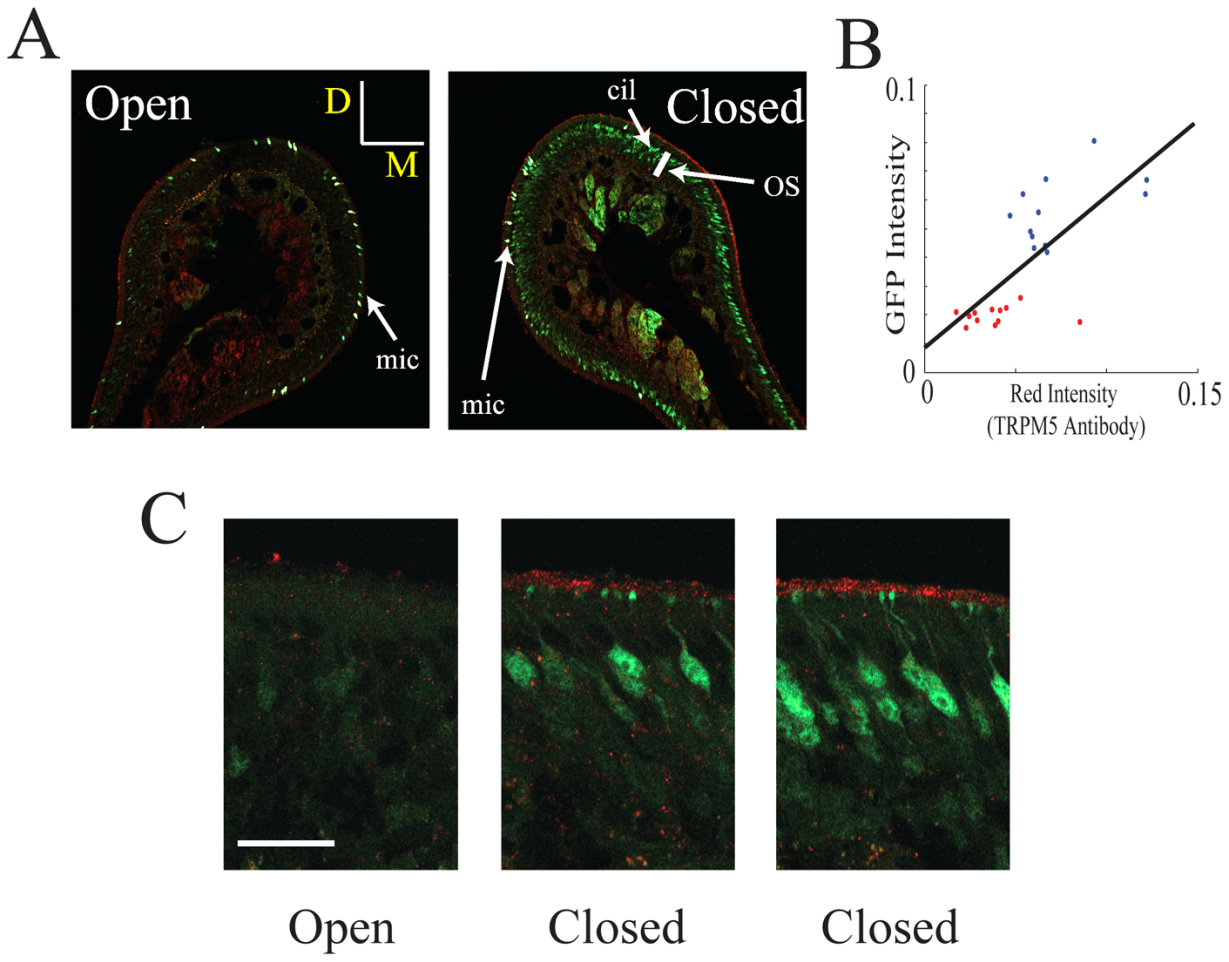
Naris occlusion upregulates the intensity of ciliary layer immunolabeling with a TRPM5 antibody. Immunolabeling for TRPM5 (red) and GFP (green) is shown in the naris open and occluded sides under two different magnifications. A. Immunolabeling in the open and closed nostrils in endoturbinate II (bar is 50 µm, D is dorsal and M is medial). Notice that there is more intense labeling for both GFP and TRPM5 in the olfactory epithelium soma layer (OS) and cilia (cil) respectively in the closed nostril and that within each turbinate the labeling was inhomogeneous (some areas in the section show higher intensity than others). In addition, as expected from our earlier study [27] there is intense labeling of microvillar cells that are not being studied here (mic). B. GFP immunolabeling intensity in the olfactory soma layer vs. TRPM5 immunolabeling intensity in the ciliary layer (red) measured in 20 degree sections around the endoturbinates in A (see the methods). Intensity in the image ranged from 0 to 1. Blue: closed, red: open. The straight line is a best fit for all the points. The correlation coefficient is 0.72 (different from zero, p<0.0001). The intensity for both GFP and TRPM5 immunolabeling in the open nostril are statistically significantly different compared to intensity in the closed nostril (t-test, p<0.000001). C. Higher magnification figures for the TRPM5 (red) and GFP (green) immunohistochemistry (bar is 20 µm, no microvillar cells are found in these images). The two images for the epithelium in the closed naris were from areas that displayed different intensity for labeling for the two antibodies.

A 14- µm-thick z-stack image of the OE was obtained using either SP5 Leica or Olympus Fluoview confocal laser-scanning microscopes. The intensity for GFP immunofluorescence in the epithelium and TRPM5 expression in the cilia was determined using MATLAB (MathWorks). Average gray scale values scaled from zero to one taken from the septum and the lateral epithelium were compared between the open and closed sides. This manuscript is focused on the expression of GFP in OSNs of TRPM5-GFP mice [16]. In addition to OSNs GFP is expressed at high intensity in a small number of microvillar cells [27]. We did not find a difference in GFP immunofluorescence in the microvillar cells when the naris occluded epithelium was compared to the naris open (not shown). Whenever possible the regions of interest (ROIs) excluded the microvillar cells. Excluding the microvillar cells was possible in the analysis shown in all figures, but the magnification in Figure 1 did not allow doing this for the analysis shown in Figure 1B and in that figure ROIs included both microvillar and OSN cells. Including microvillar cells made a minor difference and resulted in a more conservative evaluation of changes in intensity due to naris occlusion. In order to generate Figure 2B a point was defined in the middle of the endoturbinates shown in Figure 2A and a line was drawn from that point to the epithelium to define small ROIs of the epithelium (for GFP) or the ciliary layer (for TRPM5 immunohistochemistry). Each ROI encompassed a turn of this line by 20 degrees.

#### Olfactory bulb

OBs were sliced at 18 µm and mounted onto glass slides (Superfrost ® Plus, Microscope slides, Fisher Scientific). The first section was collected when the first glomerulus appeared and every section was collected until the last glomerulus disappeared from sight during the cryosectioning. The slices were divided into three groups based upon their location along the rostro-caudal axis: the rostral one-third, middle one-third, and caudal one-third. Following immunostaining, OB sections were imaged using the Nikon Eclipse E600 and analyzed in MATLAB (MathWorks). Fluorescence intensity was measured in an arbitrary scale (0–4095) that was calibrated to yield the same intensity in different sessions. Average intensity around the glomerular layer was measured and analyzed using Student's t-test between groups (naris occluded vs. untreated, WT vs. CNGA2 knockout).

### Quantitative real time PCR

#### Naris occluded mice

TRPM5-GFP mice were unilaterally naris occluded on their right nostril on PD5 and euthanized at PD58. Twenty naris occluded animals and 10 control animals were euthanized at PD58 by CO_2_. We chose PD58 because animals pass their puberty at around 8 weeks (PD56), and in the later experiment where we stimulated the OE with both putative pheromones and odorants all animals were past PD58. Tissues from two animals were pooled together in one sample, while right/left laterality was preserved during the process.

#### CNGA2 knockout mice

Five CNGA2 knockout mice and 6 control WT mice were euthanized by CO_2_. CNGA2 knockout animals were used between PD36 and 98. Control WT animals were PD41. Left and right OE from one animal were pooled together in one sample, as no naris occlusion was conducted in CNGA2 knockout mice.

#### Total RNA extraction

The OE was immediately dissected out and frozen using liquid nitrogen. The OE was homogenized in TriReagent (Sigma-Aldrich), using a Qiagen homogenizer, and total RNA was extracted using RNeasy Mini-kit (Qiagen). Following total RNA extraction, cDNA synthesis and q-RTPCR were performed in the PCR core at University of Colorado at Denver Anschutz Medical Campus. One µg total RNA was used to synthesize cDNA using High Capacity c-DNA Reverse Transcription kit (ABI-P/N 4368814). cDNA was diluted 1∶ 2 before PCR amplification. The primers for GFP were designed using Primer Express and synthesized by Applied Biosystems. The primers for TRPM5, olfactory marker protein (OMP) and Gα_olf_ were predesigned by Applied Biosystems. q-RTPCR was conducted using ABI 7900 system with 5 µl diluted cDNA. Thermal cycling conditions were as follows: Activation of TaqGold at 95°C for 10 min followed by 40 cycles of amplification at 95°C for 15 secs and 60°C for 1 min. 18 S ribosomal RNA was amplified as an internal standard. The mRNA values (pg) of TRPM5, OMP, and Gα_olf_ were normalized to 18 S rRNA values (ng) ( = normalized values)(mRNA/18 S rRNA) and the normalized values were used in the statistical analyses.

### Underwater EOG recordings

Underwater EOG recordings were made from the animal's right half of the OE perfused using Ringer's solution (145 mM NaCl, 5 mM KCl, 20 mM HEPES, 1 mM CaCl_2_, 1 mM MgCl_2_, 1 mM Na-pyruvate, 5 mM Glucose, pH 7.4 with NaOH). The EOG recordings were performed as described previously by Lin et al., (2007). Briefly, mice were euthanized by CO_2_ followed by cervical dislocation. The skull was separated down the midline, and the nasal septum was removed to expose the endoturbinates. One half of the bisected head was mounted on a recording chamber by using the dental adhesive Impregum F (ESPE, Neuss, Germany). The olfactory epithelium was continuously perfused with Ringer's solution. All recordings were made from endoturbinate II with a recording pipette with 0.9% agar (Fluka) in Ringer's. Six repetitions of recordings were made per odor. 1∶100 and 1∶200 Urine, 25 µM odorant and 1 mM 3-isobutyl-1-methylxanthine (IBMX) were freshly dissolved in Ringer's solution on the day of experiment.

The recordings were amplified with either an A-M Systems Micro electrode AC amplifier Model 1800 or a Model 3000 AC/DC differential amplifier (A-M Systems, Inc.), and digitized with Digidata 1200 (Molecular Devices). Data was digitized at 2 kHz and acquired with Clampex 8.0 software (Molecular Devices). All data were used for analysis. The perfusion system for odorant Stimulation was controlled by Clampex 8.0 with General Valve Corporation Valve Driver II. There was approximately a one second delay between opening of the valve and delivery of the odor to the epithelium. Data were analyzed using Axograph software. For the subsequent analysis, the largest peak from the six repetitions was used.

#### EOG Data analysis

The cAMP phosphodiesterase inhibitor IBMX (1 mM) was used to measure widespread activation of OSNs. To control for the number of responsive cells as well as possible run down in the response over time, the absolute amplitude (mV) in the peak EOG response to an odorant was normalized to the absolute amplitude (mV) in the peak EOG response to IBMX that was recorded 2 min after the odorant stimulation ( =  Ratio[Odor/IBMX]). Normalization of the EOG has been used in a considerable number of studies [4], [28]–[30] because it controls for the fact that in every recording site in the epithelium there is considerable variance due to: 1) The variation in the location of the electrode tip with respect to the surface of the epithelium. 2) The variation of responsiveness as a function of time. Figure S1 shows that as expected the variance of the peak EOG response elicited by an odor after normalization to the IBMX peak EOG response is significantly smaller than the variance of the peak EOG response to an odor not normalized to IBMX.

### Statistical analysis

Student's t-test or ANOVA were used to test for significance of difference between groups. For t-test that was used for comparing more than two samples we used false discovery rate (FDR) for correcting for multiple comparisons [31].

## Results

### GFP immunofluorescence in OSNs from TRPM5-GFP mice is increased upon unilateral naris occlusion

A subset of OSNs expresses GFP in an adult TRPM5-GFP mouse where the TRPM5 promoter drives GFP expression [16], [32]. Those OSNs express OMP (olfactory marker protein), a marker of mature OSNs [16]. Previous studies have shown that in the occluded side of epithelia, the expression of the OMP, ACIII and CNGA2 was increased [20]–[22], [33]. Whether the expression of TRPM5 is increased by unilateral naris occlusion was unknown. Here we show that the GFP immunofluorescence in TRPM5-GFP animals is increased in the occluded side of the epithelia. We performed unilateral naris occlusion at PD5 and examined GFP immunofluorescence 1 month later (Figures 1 and 2). Figure 1A shows a 14 µm coronal section of mouse OE that underwent unilateral naris occlusion, leaving one nostril intact to serve as an internal control. In the image, the left half is the open side (control) and the right half is the closed side (occluded). In the open side, GFP is highly expressed in the lateral and ventral regions, consistent with observations made in the untreated animals from a TRPM5-GFP line in the previous study by Lin and colleagues [16]. We found that GFP immunofluorescence was significantly increased in the occluded side of the epithelia compared to the open side (Figure 1A, right side). Average intensities across the left/right sides in the septum, and in the left/right lateral regions were measured. Average intensity measured from the septum (dorsal to ventral: i, ii and iii), and lateral OE (iv) regions are shown in the bar graphs (Figure 1B). We found that the occluded side of both the septum and the lateral OE showed significantly higher expression of GFP compared to the control sides, and the effect was consistent across all animals tested (i; *p* = 0.02, ii; *p* = 0.006, iii; *p* = 0.002, iv; *p* = 0.007, p value FDR corrected, *p* = 0.05, paired t-test, n = 4). This evidence indicates that TRPM5 expression is increased by naris occlusion.

### TRPM5 expression in the ciliary layer is increased upon unilateral naris occlusion

Figure 2 shows that naris occlusion results in a substantial increase in the intensity of TRPM5 immunolabeling in the olfactory ciliary layer and that in the same epithelium immunohistochemistry for GFP driven by the TRPM5 promoter in the OSNs was also increased. The increase in green fluorescence reflects an increase in the number of OSNs expressing GFP fluorescence. In contrast the intensity of ciliary immunostaining for the Ca^2+^ activated Cl^−^ channel ANO2 [10], [34] is not altered by naris occlusion (Figure S2). The intensity of immunostaining for GFP and TRPM5 were directly related with a correlation coefficient of 0.72 (different from zero, p<0.0001, Figure 2B). Interestingly, the immunolabeling for TRPM5 and GFP was inhomogeneous along different areas of the epithelium (Figure 2A). Indeed, even within areas of the olfactory epithelium of TRPM5-GFP mice where some soma were strongly labeled with the GFP antibody a subset of OSN cell bodies displayed relatively low GFP immunofluorescence (Figure 2C).

### Unilateral naris occlusion increases GFP immunofluorescence in axons innervating glomeruli in TRPM5-GFP mice

**Figure 3:**
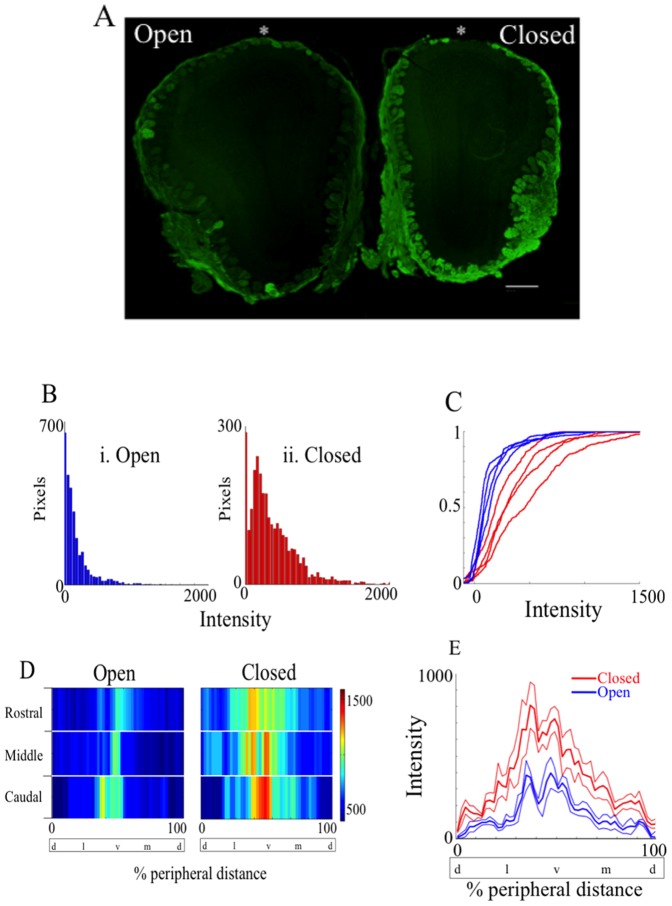
Naris occlusion upregulates GFP presence in the OB. A shows the representative coronal 18 µm section of the naris-occluded OB of a TRPM5 GFP animal (scale bar = 500 µm). The right OB in the image is ipsilateral to the occluded (Closed) naris, and the left OB is ipsilateral to the open naris. The section was immunostained with an antibody against GFP (green). As expected, the OB is smaller in the occluded side [52]. (B) Histogram of the number of pixels as a function of the fluorescence intensity (0–4095) after subtracting intensity taken from the external plexiform layer (EPL) just underneath the glomerular layer. GFP immunofluorescence is higher in the occluded side (ii) compared to open side (i). (C) Cumulative histogram for fluorescence intensity after subtraction of EPL fluorescence levels for all four animals examined. Occluded OBs (red) express GFP at significantly higher level than open OB (blue) (t-test for mean intensity, *p* = 0.0286, n = 4). D shows a 2D color map of the glomeruli displaying GFP immunofluorescence as a function of percentage distance from the dorsal most point (*) around the glomerular layer in the olfactory bulb in A. Three representative OB sections were taken from the rostral, medial and caudal one-third and analyzed for GFP immunofluorescence intensity around the glomerular layer. (E) Mean fluorescence intensity around the glomerular layer of the occluded (red) and the open OB (blue). Thin lines represent the standard error of the mean (SEM). The intensity was averaged for caudal, middle and rostral images. Occluded side of OB (red) significantly differs from the open side of the OB (blue) (*p*<0.0001, N-Way ANOVA, n = 4). % peripheral distance was measured starting from the dorsal most point. d = dorsal, l = lateral, v = ventral, m = medial.

Axons of OSNs expressing the same olfactory receptor converge onto one or two spherical neuropil called glomeruli in the OB [35], [36]. A previous study by Lin and colleagues (2007) showed that in TRPM5-GFP animals GFP was transported to the axon terminals of the OSNs within the glomeruli in the OB and could be imaged using an antibody against GFP. We reasoned that if there was an increase in GFP immunofluorescence in the main olfactory epithelia there would be an increase in GFP immunofluorescence in the glomeruli in the OB because of transport of the GFP down the OSN axons. Figure 3A shows a representative coronal section of the OB of a naris occluded TRPM5-GFP animal. In the image, the right bulb is ipsilateral to the occluded naris (closed), and the left bulb is ipsilateral to the open (open) side of naris. Three representative sections from the rostral, middle, and caudal one-third were taken from four animals. The OB sections were immunoreacted with an antibody against GFP with a secondary fluorescent antibody and fluorescent images were acquired. Fluorescence intensity around the glomerular layer was measured and analyzed. Figure 3B shows histograms of the number of pixels as a function of fluorescence intensity (0–4095). For this figure, the intensity taken from the external plexiform layer (EPL) just underneath the glomerular layer was subtracted from the intensity taken from the glomerular layer. In the occluded side of OB (ii, red), a number of pixels reside within the higher range of intensity values, whereas in the open bulb (i, blue) a majority of pixels are concentrated within the lower range of intensity values. A cumulative histogram for all four animals tested (Figure 3C) shows a significant difference in glomerular fluorescence intensity between the open (blue) and occluded sides (red) of the OBs (t-test for mean intensity, *p* = 0.0286, n = 4).

Fluorescent intensity around the glomerular layer of the OB was plotted in a two dimensional color map as a function of the percent peripheral distance from the dorsal most point around the transverse section (i.e. the dorsal most point to the dorsal most point =  100%) (Figure 3D). The glomeruli displaying GFP immunofluorescence tended to be located at the ventral OB (ventral = 50% in percentage distance) in the control side of OB, consistent with previous studies [16], [37]. In the occluded side of the OB however, the intensity is significantly increased around the ventral regions, and there appears to be a broadening of the GFP immunofluorescence, implying a broad increase in GFP expression in the OSNs in the occluded side of OB. Middle OB slices were chosen to quantify differences in GFP intensity around the glomerular layer between occluded and control sides of the OB (Figure 3E). Figure 3E shows the mean fluorescence intensity around the glomerular layer measured from closed (red) and open (blue) OB as a function of the percentage peripheral distance (%) from the dorsal most point. Consistent with the 2D color map in Figure 3D we found that GFP intensity is broadly increased regardless of the location from the dorsal most point, suggesting that the expression of GFP may not be restricted to a subpopulation of the OSNs, but rather a majority of OSNs express GFP under TRPM5 promoter, and that GFP immunofluorescence is affected by naris occlusion (two way ANOVA for open vs. closed naris *F*_(1,49)_ = 240, *p*<0.0001, n = 4).

### High fluorescence intensity for glomerular expression of GFP in TRPM5-promoter-driven GFP immunofluorescence in CNGA2 knockout mice

Unilateral naris occlusion is an accepted method to reduce olfactory sensory input, however, changes can be due to other effects such as a change in air pressure during sniffs or changes in pH due to differences in CO_2_ between open and closed nares. We reasoned that if an increase in GFP expression in TRPM5-GFP mice is a result of decreased odor-induced activity of OSNs, we would see an increase in GFP immunofluorescence in the glomeruli in mice defective for subunit A2 of the cyclic nucleotide-gated channel that mediates odor transduction in OSNs (CNGA2 knockout mice). The CNGA2 knockout mouse line is known to be largely anosmic as recorded EOG amplitudes in the OE of CNGA2 knockout mice are significantly decreased or not detectable in response to odorants [23], [24].

**Figure 4:**
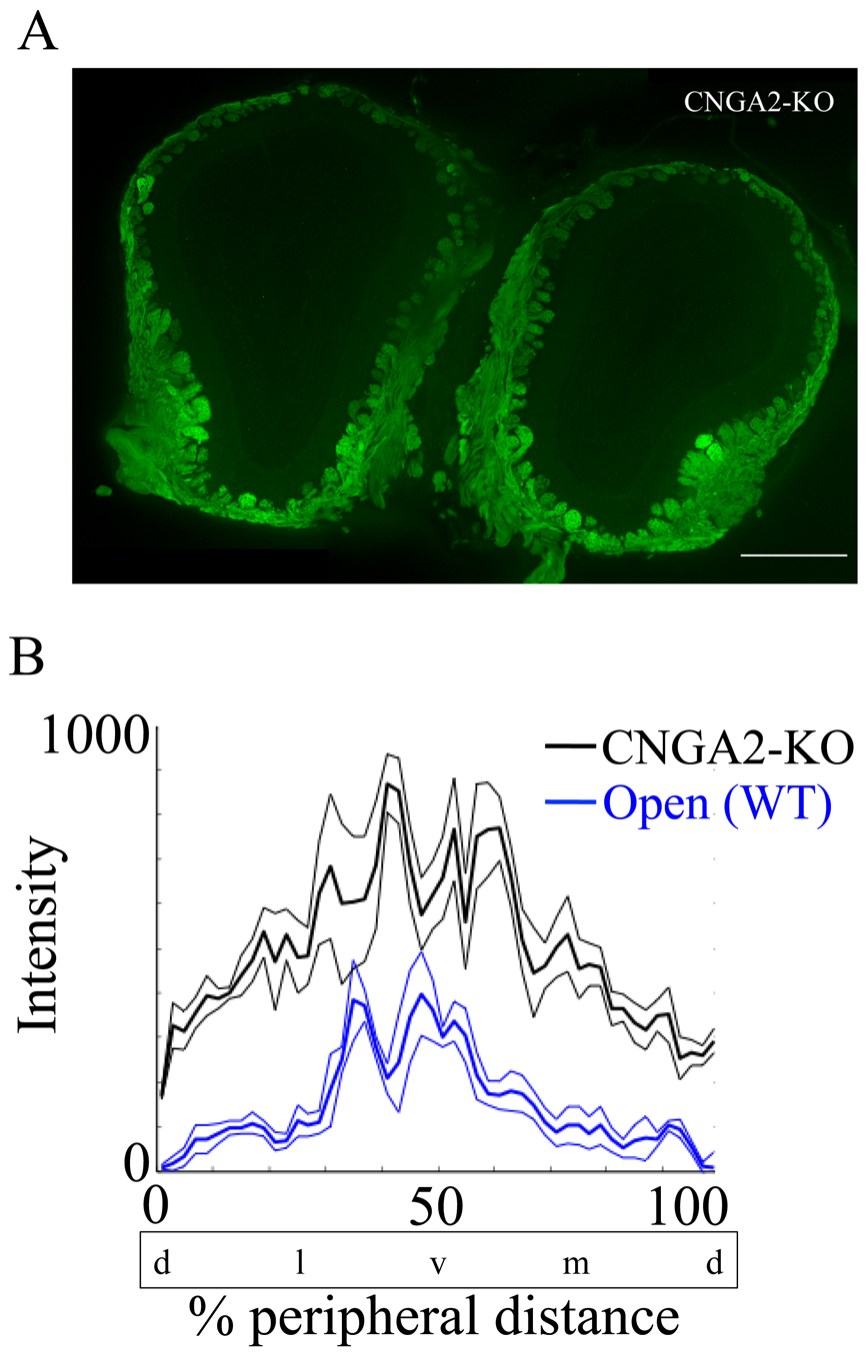
CNGA2 knockout OB shows wider distribution of glomeruli displaying GFP immunofluorescence that differs from the WT OB. A shows a representative coronal section of the OB of CNGA2 knockout/TRPM5-GFP mouse (CNGA2 KO in the figure). Eighteen µm OB section was immunoreacted with antibody against GFP (green, scale bar = 500 µm). B shows the mean fluorescence intensity around the glomerular layer as a function of percent peripheral distance from the dorsal most point. Thin lines represent the SEM. The CNGA2 knockout OB (black) significantly differs from the open side of naris occluded OB (blue) of TRPM5-GFP animals (Open in the figure). (two way ANOVA for CNGA2 knockout/TRPM5-GFP vs. TRPM5-GFP open naris *F*_(1,49)_ = 189, *p*<0.0001, n = 4–6). % peripheral distance was measured starting from the dorsal most point. d = dorsal, l = lateral, v  =  ventral, m = medial.

Figures 4 and S3 show GFP immunofluorescence in the OB and OE of CNGA2 knockouts and wild type mice. Figure 4A shows a representative coronal section of the olfactory bulbs of a CNGA2 knockout/TRPM5-GFP mouse immunoreacted with an antibody against GFP. Sections from the middle one-third were analyzed for GFP intensity around the glomerular layer. In Figure 4B mean fluorescence intensities around the glomerular layer of CNGA2 knockout OB (black) and the open side of the OB of naris occluded TRPM5-GFP animals (blue) were plotted as a function of the percentage peripheral distance from the dorsal most point. Strikingly, we found that GFP immunofluorescence around the glomerular layer is more intense in CNGA2 knockout/TRPM5-GFP compared to the open side of the OB of TRPM5-GFP animals. The GFP intensities around the glomerular layer (Figure 4B) significantly differ between the CNGA2 knockout/TRPM5-GFP OB and open side of the OB of naris occluded TRPM5-GFP animals (two way ANOVA for CNGA2 knockout/TRPM5-GFP vs. TRPM5-GFP open naris *F*_(1,49)_ = 189, *p*<0.0001, n = 4–6). GFP immunofluorescence was also more widely distributed along the angle around the olfactory bulb slice in the CNGA2 knockout/TRPM5-GFP compared to the open side of the TRPM5-GFP mouse as shown by the fact that even after the fluorescence intensity was normalized to the ventral GFP intensity the distribution tested by a two way ANOVA differed between CNGA2 knockout/TRPM5-GFP and TRPM5-GFP (not shown). This evidence suggests that the lack of odor-evoked activity due to the genetic deletion of CNGA2 leads to an elevated expression of glomerular GFP under TRPM5 promoter activity.

### Higher expression of TRPM5 mRNA in the naris occluded side of the OE and in CNGA2 knockout OE

Unilateral naris occlusion has been shown to regulate protein and mRNA expression in OSNs [20]–[22], [33]. OMP, is a 19-kD cytosolic protein, which is thought to be involved in regulating the time course of the odor response [38], [39]. Gα_olf_ is the G-protein mediating olfactory signal transduction [3]. Both OMP and Gα_olf_ are expressed only by mature OSNs. OMP and Gα_olf_ expression in the occluded MOE becomes significantly denser compared to the open side as early as 11 days post-naris occlusion [20], [33].

**Figure 5:**
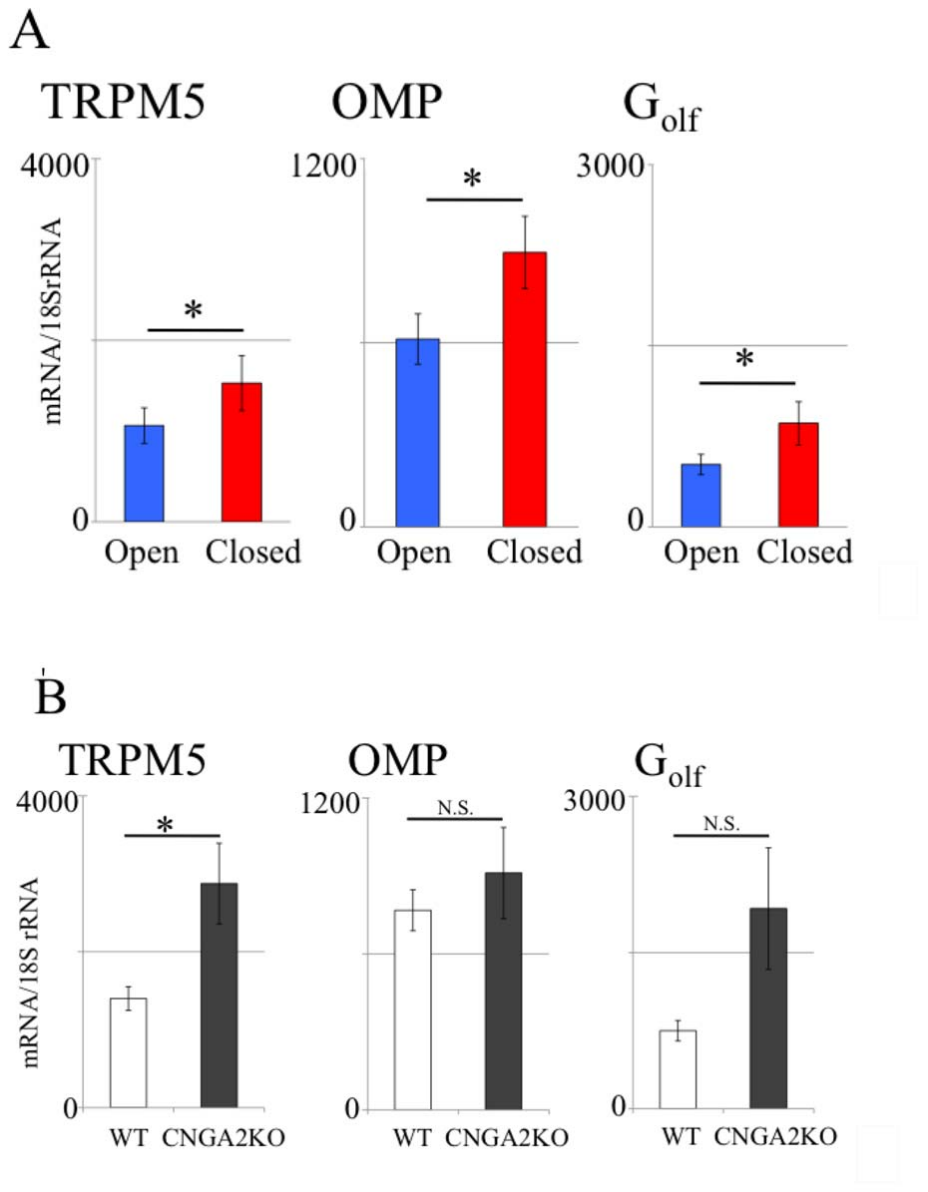
Decreased odor-evoked OSNs activity leads to an elevated level of TRPM5 mRNA. The mRNA levels of TRPM5, OMP, and Gα_olf_ were compared between: A. left (open) and right (closed, occluded) OE of the naris-occluded TRPM5-GFP animals. B. CNGA2 knockout/TRPM5-GFP and TRPM5-GFP control OE. mRNA levels are normalized to levels of 18 S rRNA. In the occluded OE, there was significantly higher expression of mRNA of TRPM5 (*p* = 0.024, n = 10), OMP (*p* = 0.008, n = 10), and Gα_olf_ (*p* = 0.033, n = 10, paired t-test, p value FDR corrected *p* = 0.05).Untreated control animals showed no significant difference between left and right OE (*p*>0.05, paired t-test, n = 5, data not shown). The mRNA expression level of TRPM5 was significantly higher in CNGA2 knockout/TRPM5-GFP compared to TRPM5-GFP (*p* = 0.016, unpaired t-test, p value FDR corrected *p* = 0.0167, n = 5). There was no significant difference between CNGA2 knockout/TRPM5-GFP and TRPM5-GFP in mRNA expression levels for OMP and Gα_olf_ (*p*>0.05, unpaired t-test FDR corrected, n = 5). Error bars are SEM.

We tested whether mRNA expression levels of TRPM5, OMP and Gα_olf_ expressed by OSNs (normalized to 18S rRNA expression levels) is changed by naris occlusion (Figure 5). We found that in the naris-occluded TRPM5-GFP animals, the occluded side of OE (red) had significantly higher expression of mRNA compared to open side of OE (blue) for TRPM5 (*p* = 0.0237, n = 10), OMP (*p* = 0.0076, n = 10), and Gα_olf_ (*p* = 0.0329, n = 10, paired t-test, FDR corrected p value = 0.05). In untreated control animals, there was no significant difference in mRNA normalized expression level between left and right OE for all three genes (paired t-test, n = 5, data not shown). The increase in OMP normalized mRNA level observed in the present study is consistent with the study by Coppola and colleagues where they showed an increased immunoreactivity against OMP in the OE when they compared the occluded side to the open side [20] and the increase in Gα_olf_ is consistent with a study by He and co-workers [33]. In addition, we examined whether the OE of CNGA2 knockout/TRPM5-GFP had increased normalized mRNA expression of TRPM5, similar to the elevated GFP immunofluorescence we observed in the open side of the OB of naris occluded TRPM5-GFP animals (see Figure 5). We found that the normalized mRNA expression level of TRPM5 was significantly higher in CNGA2 knockout/TRPM5-GFP compared to TRPM5-GFP (*p* = 0.0159, n = 5). There was no significant difference between CNGA2 knockout/TRPM5-GFP and TRPM5-GFP in normalized mRNA expression level of OMP (*p*>0.05, n = 5) and Gα_olf_ (*p*>0.05, n = 5, unpaired t-test, FDR corrected p value  = 0.0167), indicating that normalized TRPM5 mRNA expression is elevated in CNGA2 knockout OE, implying that TRPM5 may have an important role in the OSNs with decreased odor-evoked activity.

### TRPM5 is involved in increasing/maintaining odor responses for a subset of putative pheromones in the occluded side of the epithelium

In this study, we show that the TRPM5 expression is increased in the occluded side of the epithelium. A study has shown that unilateral naris occlusion increased air EOG amplitude in the occluded side of the epithelium, possibly due to the increased expression of the transductory proteins [40]. Thus we decided to ask whether TRPM5 is involved in mediating the olfactory transduction for the putative pheromones in the occluded sides of the epithelia. In this set of experiments, we recorded *underwater* EOG responses from four different animal groups, which were untreated TRPM5-GFP (TRPM5(+), untreated), naris occluded TRPM5-GFP (TRPM5(+), naris occluded), untreated TRPM5 knockout/TRPM5-GFP(TRPM5(-), untreated), and naris occluded TRPM5 knockout/TRPM5-GFP (TRPM5(-), naris occluded). The naris occluded TRPM5(+) and TRPM5(-) mice received unilateral naris occlusion on their right nostril at PD5.

**Figure 6:**
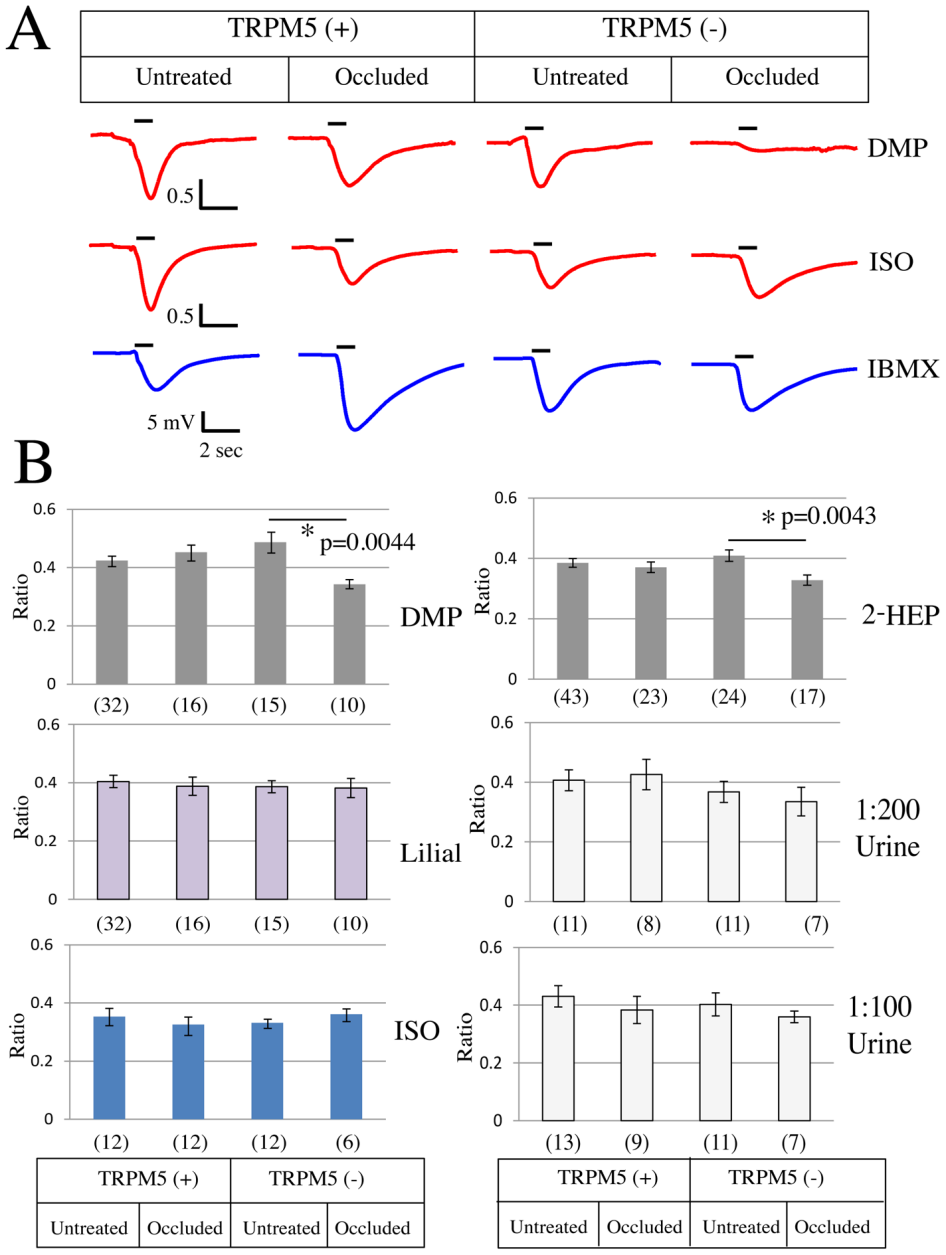
Underwater EOG response to DMP and 2-heptanone was affected by unilateral naris occlusion in TRPM5(-) epithelia. A; Example traces of EOG. The responses to DMP and isoamylacetate (ISO) are depicted in red and the response to IBMX is depicted in blue. The black bars above the traces indicate when the stimuli reach the epithelia and the duration of the stimulus application ( = 1 second). B; Putative pheromones (2,5-dimethylpyrazine (DMP) and 2-heptanone (2-HEP)), diluted urine (1∶200 or 1∶100) and general odorants (lilial and isoamylacetate) were tested for underwater EOG. What is reported is the ratio of the response to the pheromone/odorant divided by the response to IBMX [odor/IBMX]. In TRPM5(+) olfactory epithelia, naris occlusion had no effect on the ratio [odor/IBMX] (p value>0.05, paired t-test, FDR corrected). However, in TRPM5(-) olfactory epithelia, naris occlusion significantly reduced the ratio [odor/IBMX] to 2-heptanone or DMP (p<0.01, paired t-test, FDR corrected). The numbers in parentheses show the number of epithelia tested. The error bars are SEM.

As indicated in the methods to control for the number of responsive cells as well as possible run down in the response over time, the absolute amplitude (mV) in response to an odorant was normalized to the absolute amplitude (mV) in response to IBMX that was recorded 2 min after the odorant stimulation ( =  Ratio[Odor/IBMX]). Figure 6 summarizes the EOG results recorded by using 5 different stimuli. Lilial and Isoamylacetate (Figure 6B) were tested as general odorants that activate a large proportion of OSNs in the main olfactory epithelia. DMP (2,5-dimethylpyrazine) and 2-heptanone (2-HEP) were tested as putative pheromones that were shown to activate a small subset of glomeruli in CNGA2 knockout mice [16]. Urine was tested as an environmental odor that animals are most exposed to in their home cage. The ratio [Odor/IBMX] was compared between untreated epithelia and occluded epithelia. We found that in TRPM5(+) group there was no significant difference between the untreated (untreated) and the occluded epithelia (naris occluded) regardless of the odorant used (Figure 6B,C). However, in TRPM5(-), group the ratio [odor/IBMX] in response to DMP (paired t-test, p = 0.0044, FDR corrected) and to 2-Heptanone (paired t-test, p = 0.0043, FDR corrected) was significantly lower in the occluded epithelia (naris occluded) compared to the untreated epithelia (untreated), suggesting that under a decreased olfactory input, TRPM5 has a role in the olfactory transduction of the putative pheromones.

## Discussion

In this study, we demonstrated that TRPM5 expression in OSNs is increased by unilateral naris occlusion and that mice that have CNGA2 genetically deleted show elevated expression of TRPM5. In a complementary experiment EOG odor responses showed that TRPM5 plays a role in maintaining pheromone responsiveness after naris occlusion. Evidence has suggested that the main olfactory system also processes pheromones along with the vomeronasal system [14], [41], [42] and a subset of main olfactory bulb olfactory glomeruli [43], including some of the TRPM5-GFP glomeruli [37], are innervated by mitral cells that send axons to the vomeronasal amygdala. In the present study, the potential involvement of TRPM5 in sensing pheromones during reduced olfactory input was investigated.

### Unilateral naris occlusion increases TRPM5 expression in the occluded OE

Studies have shown that unilateral naris occlusion upregulates transductory mRNA and proteins in the occluded OE [20], [22], [33], [40]. It has been hypothesized that by upregulating the transduction proteins animals are compensating their lack of olfactory responsiveness to odorants. We reasoned that if TRPM5 is involved in odor transduction there might be a substantial increase in TRPM5 expression as found for proteins involved in transduction. Strikingly, in TRPM5-GFP mice unilateral naris occlusion caused a substantial increase in GFP expression in a large population of the OSNs that express GFP at low levels in the open naris (Figures 1, S2 and 2). In addition, the immunostaining for TRPM5 in the olfactory cilia increased upon naris occlusion and the intensity of GFP immunofluorescence in the olfactory soma layer of the OE was directly related to the intensity of TRPM5 immunostaining in the ciliary layer (Figure 2). The findings in the olfactory epithelium were consistent with a large increase of GFP immunofluorescence in glomeruli (Figure 3). Finally, naris occlusion elicited a significant increase in expression of TRPM5 mRNA (Figure 5). The magnitude of the increase in TRPM5 mRNA was smaller than the magnitude of increase in GFP immunofluorescence or TRPM5 immunostaining but this likely due to the expression of TRPM5 mRNA in the microvillar cells [44] whose GFP immunofluorescence was not changed upon naris occlusion (not shown). The microvillar cells do not send axons to the OB and therefore their presence does not affect GFP immunofluorescence in the OSN axons and OB glomeruli.

### Wider ventral expression of TRPM5 in the olfactory bulb of CNGA2 knockout mice

Next we asked whether lack of odor-evoked activity caused via the genetic deletion of CNGA2 would increase TRPM5 expression in OSNs whose axons reach specific areas of the olfactory bulb. Although unilateral naris occlusion is an accepted method to effectively reduce olfactory sensory input, it has other deleterious effects on the olfactory epithelia. Studies have reported that following naris occlusion there was decreased metabolism [45], neurogenesis and the number of proliferating immature cells [46], [47], resulting in a thinning of the OE [46]. In addition, the possible changes in pH, air pressure, temperature, humidity and stress-responding protein expression in the occluded nasal cavity and the effect of those changes on the TRPM5 expression could not be excluded as possible confounding factors. Crossing CNGA2 knockout mice with TRPM5-GFP mice allowed us to examine the effect of olfactory sensory reduction on TRPM5 expression without changing the factors listed above. We found that in CNGA2 knockout/TRPM5-GFP mice the glomeruli displaying GFP immunofluorescence had a wider spatial distribution in the ventral region and that the GFP intensity around the glomerular layer as a function of the percentage peripheral distance from the dorsal most point of the CNGA2 knockout mice was increased compared to the open OB of WT naris occluded mice, suggesting that the lack of CNGA2-mediated, odor-evoked activity leads to an increased TRPM5 expression in the OSNs. In CNGA2-GFP knockout mice a small subset of GFP expressing glomeruli were activated by putative pheromones, evidenced by increases in c-Fos expression in PG cells [24], which implied a presence of an alternative transduction pathway in the absence of functional CNG channels. Here we show that, consistent with an involvement of TRPM5 in putative pheromone transduction in OSNs in CNGA2 knockout OSNs, there is an increase in TRPM5 expression in CNGA2 knockout mice compared to controls. Interestingly, the number of glomeruli displaying GFP immunofluorescence in TRPM5-GFP mice increased in the naris occluded side implicating that TRPM5 expression is increased in a subset of glomeruli that do not express substantial TRPM5 in the un-occluded side.

Importantly previous studies have shown that the olfactory epithelium of CNGA2 knockout mice shows markedly reduced EOG responses to odors [23], [24]. Taken together with the fact that here we show substantial increases in the expression of TRPM5 in CNGA2 knockout OSNs these studies raise the question whether CNGA2 is necessary to activate TRPM5. This will have to be addressed in future studies.

### TRPM5 is involved in activity-dependent plasticity of OSN responses to a subset of pheromones

What would be the functional implication of increasing expression of TRPM5 in a subset of the OSNs in the naris-occluded side? In order to determine whether TRPM5 is involved in regulating odor responses under naris occlusion we tested the effect of naris occlusion on odor and putative pheromone-induced underwater EOGs on wild type and TRPM5 knockout mice. Strikingly, when tested with the putative pheromones DMP and 2-heptanone, the EOG response showed a significant decrease caused by naris occlusion in TRPM5 knockout mice, but not in wild type mice (Figure 6C). Interestingly, TRPM5 knockout did not show any change in the EOG response caused by naris occlusion when tested either with the odors lilial or isoamylacetate, which have been demonstrated to activate the cAMP mediated olfactory transduction pathway. In addition, the EOG response to urine was also not affected by naris occlusion. Why the EOG elicited by urine, that carries pheromones, was not affected by naris occlusion is not clear, but likely is due to the fact that urine includes also a large number of general odorants[48]. Because of this the EOG that would be a response to the general odorants and the pheromones may not be affected to a statistically determined difference. Future studies with isolated OSNs are necessary to understand this result. These results are consistent with involvement of TRPM5 in a transduction pathway for responses to putative pheromones.

Our results are consistent with the findings that unilateral naris occlusion increased the expression at the mRNA and protein levels of ACIII and CNGA2 [3], [22], [33], [49], key proteins in olfactory transduction whose increased expression is likely to result in increased odor responsiveness. In addition, these investigators found that the EOG [40] and loose patch [33] odor response was larger in the occluded OE, suggesting that changes in transduction-related proteins tend to make the OE more responsive to odorants. In our studies we did not find an increase in EOG responses in the occluded OE. The difference between our study and the study by Waggener and Coppola (2007) could be due to factors such as differences in animal housing facilities [50], the difference in methods (in our study the EOG was performed with delivery of the odor in Ringer's while in theirs they delivered the odor in air), or the difference in odorant concentration used (He et al., 2012). Moreover, a study by Angely and Coppola provided behavioral evidence that showed that occluded mice with re-opened nostril paired with contralateral bulbectomy performed better compared to bulbectomy-only controls [51]. Thus, evidence including data presented here indicates that upon naris occlusion changes in gene expression tend to increase or prevent a decrease in odor responsiveness of the olfactory system. Our experiments suggest that a role of increases in the expression of TRPM5 is to increase/maintain odor responses for a subset of putative pheromones.

## Supporting Information

Figure S1 **The variance for the peak of the EOG responses to odors becomes smaller when normalized to IBMX.** A. Histograms for peak EOG response to an odor without normalization (blue) and normalized to the peak EOG response to IBMX (red). For each experiment the average of the peak EOG response was made equal to one to view the variance of the response. The results are shown for three stimuli: DMP (left), 2-heptanone (middle) and lilial (right). B. The variances were calculated from the histograms in A and are shown here in a bar graph. A two-sample F test shows a significant difference in variance between IBMX normalized vs. not normalized EOG peak responses for all odors (*, p<0.001). EOG traces are shown in Figure 6.
(TIF)

Figure S2 **Naris occlusion elicits increased immunostaining for GFP in the epithelium of a TRPM5-GFP mouse (green) but does not alter the intensity of ciliary immunohistochemistry for ANO2 (red).** This image was taken at the level of endoturbinate III. The bar is 50 µm.
(TIF)

Figure S3 **Representative coronal sections of the olfactory epithelium in CNGA2 knockouts (A) and wild type mice (B) expressing GFP under the control of the TRPM5 promoter.** A. CNGA2-KO/TRPM5-GFP olfactory epithelium. B. TRPM5-GFP olfactory epithelium. The white bar is 0.5 mm. The area in the white square is shown at higher magnification in the three images on the right of each figure. Green: GFP, Red: olfactory marker protein (OMP). Both confocal images were taken under the same laser intensity.
(TIF)
