# Supplementary figures and images for: Potential Role of Transient Receptor Potential Channel M5 in Sensing Putative Pheromones in Mouse Olfactory Sensory Neurons
Source: PLoS One. 2013 Apr 16;8(4):e61990. doi: 10.1371/journal.pone.0061990 (PMC3628705; doi:10.1371/journal.pone.0061990)

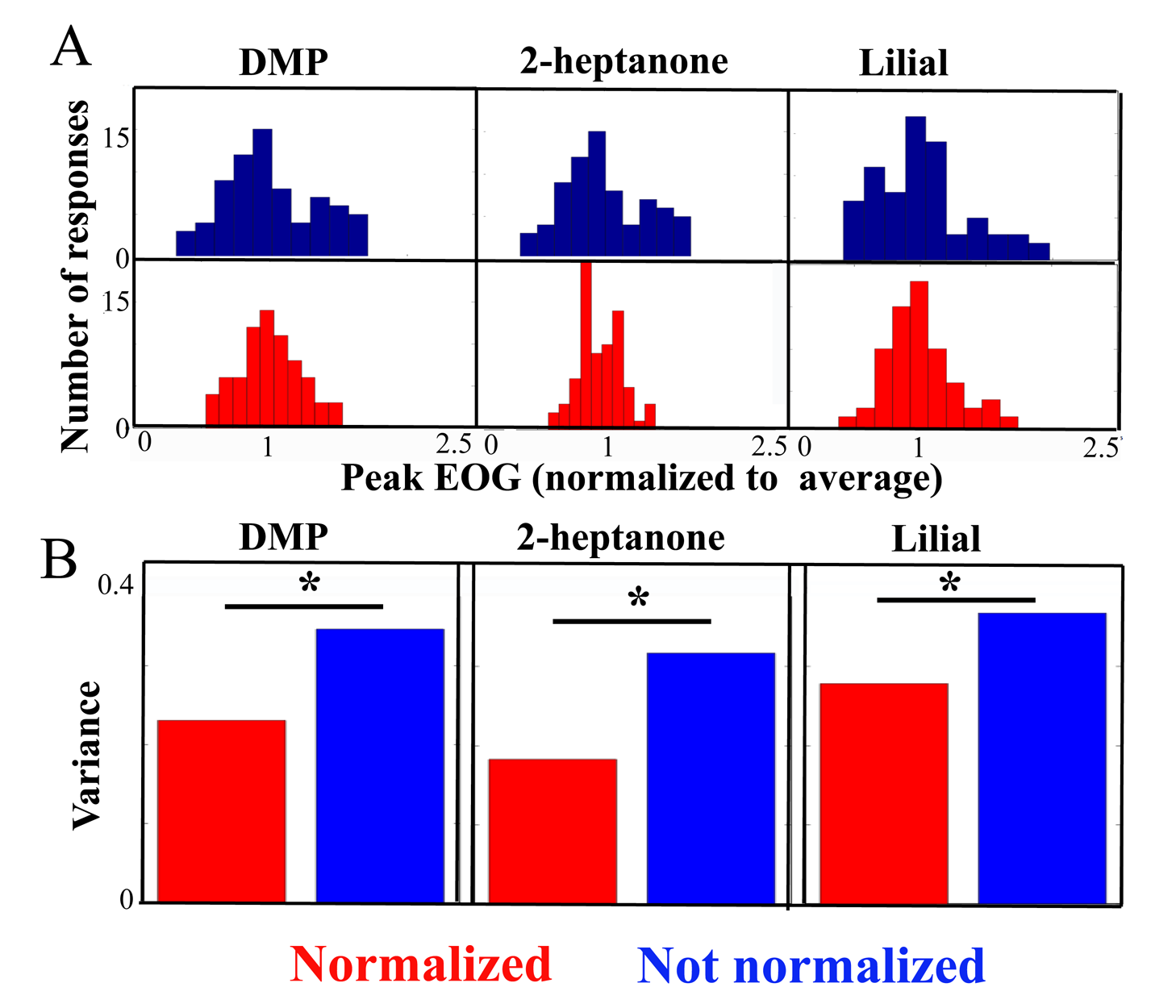

Supplement: Figure S1 — The variance for the peak of the EOG responses to odors becomes smaller when normalized to IBMX. A. Histograms for peak EOG response to an odor without normalization (blue) and normalized to the peak EOG response to IBMX (red). For each experiment the average of the peak EOG response was made equal to one to view the variance of the response. The results are shown for three stimuli: DMP (left), 2-heptanone (middle) and lilial (right). B. The variances were calculated from the histograms in A and are shown here in a bar graph. A two-sample F test shows a significant difference in variance between IBMX normalized vs. not normalized EOG peak responses for all odors (*, p<0.001). EOG traces are shown in Figure 6. (TIF) [file pone.0061990.s001.tif]

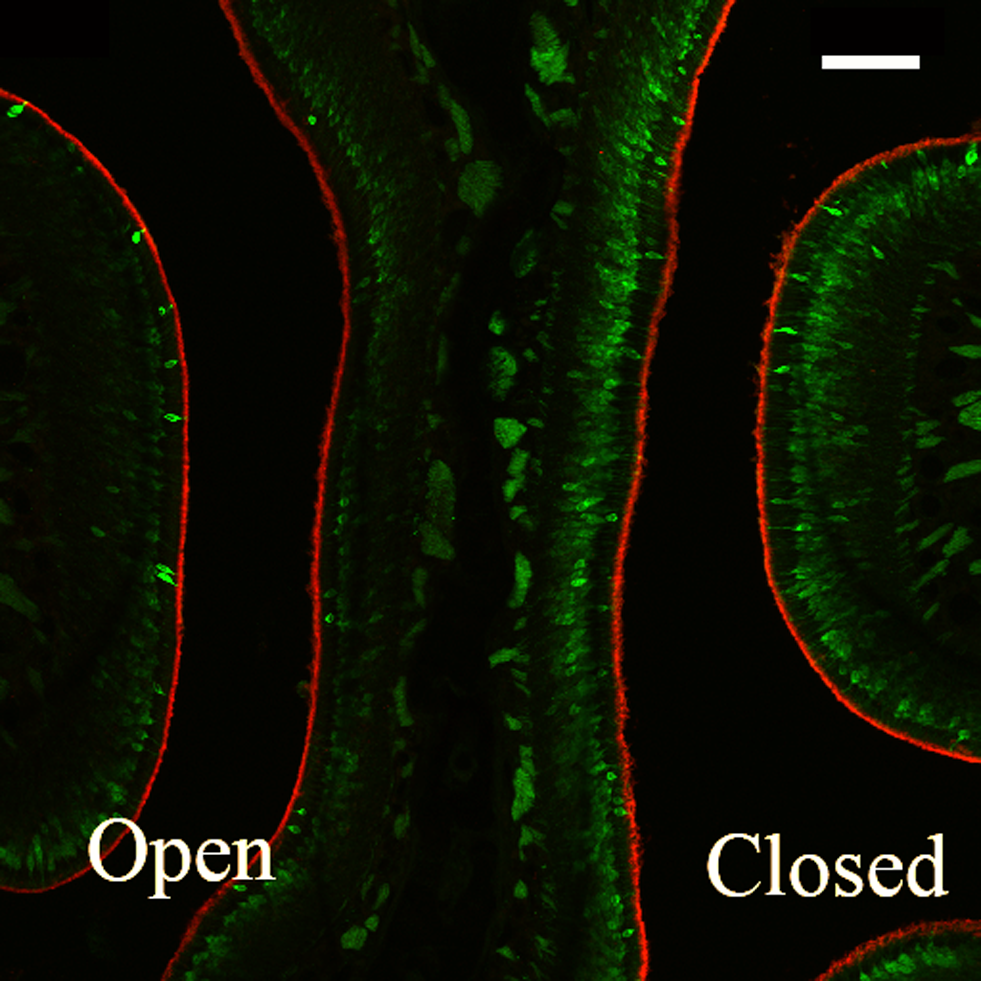

Supplement: Figure S2 — Naris occlusion elicits increased immunostaining for GFP in the epithelium of a TRPM5-GFP mouse (green) but does not alter the intensity of ciliary immunohistochemistry for ANO2 (red). This image was taken at the level of endoturbinate III. The bar is 50 µm. (TIF) [file pone.0061990.s002.tif]

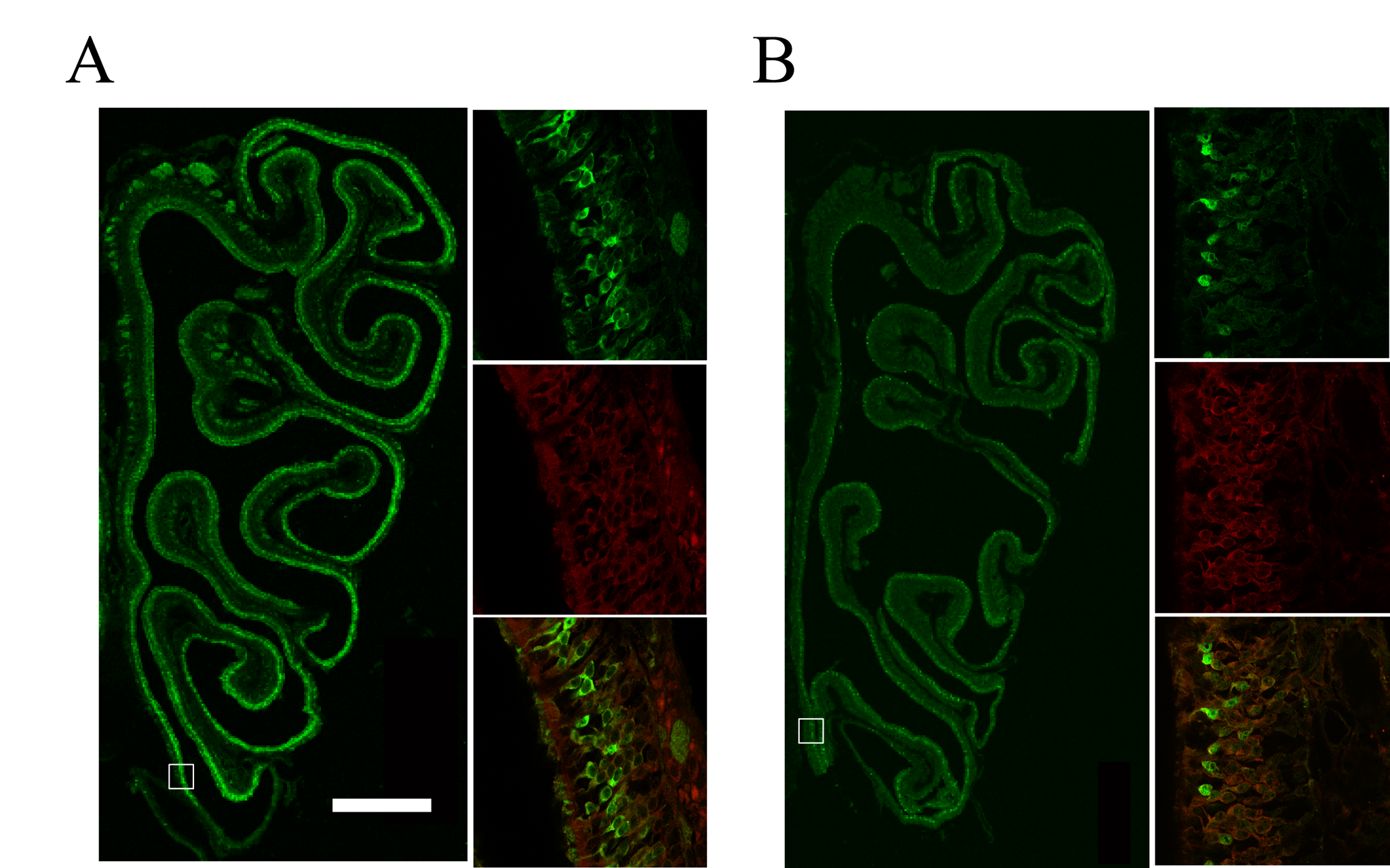

Supplement: Figure S3 — Representative coronal sections of the olfactory epithelium in CNGA2 knockouts (A) and wild type mice (B) expressing GFP under the control of the TRPM5 promoter. A. CNGA2-KO/TRPM5-GFP olfactory epithelium. B. TRPM5-GFP olfactory epithelium. The white bar is 0.5 mm. The area in the white square is shown at higher magnification in the three images on the right of each figure. Green: GFP, Red: olfactory marker protein (OMP). Both confocal images were taken under the same laser intensity. (TIF) [file pone.0061990.s003.tif]
